# Four Aromatic Sulfates with an Inhibitory Effect against HCV NS3 Helicase from the Crinoid *Alloeocomatella polycladia*

**DOI:** 10.3390/md15040117

**Published:** 2017-04-11

**Authors:** Idam Hermawan, Atsushi Furuta, Masahiro Higashi, Yoshihisa Fujita, Nobuyoshi Akimitsu, Atsuya Yamashita, Kohji Moriishi, Satoshi Tsuneda, Hidenori Tani, Masamichi Nakakoshi, Masayoshi Tsubuki, Yuji Sekiguchi, Naohiro Noda, Junichi Tanaka

**Affiliations:** 1 Department of Chemistry, Biology and Marine Science, University of the Ryukyus, Nishihara, Okinawa 903-0213, Japan; hermawan_idam@yahoo.com (I.H.); higashi@sci.u-ryukyu.ac.jp (M.H.); 2 Department of Life Science and Medical Bioscience, Waseda University, 2-2 Wakamatsu-cho, Shinjuku-ku, Tokyo 162-8480, Japan; atsushi.5961@ruri.waseda.jp (A.F.); stsuneda@waseda.jp (S.T.); 3 Biomedical Research Institute, National Institute of Advanced Industrial Science and Technology, 1-1-1 Higashi, Tsukuba, Ibaraki 305-8566, Japan; y.sekiguchi@aist.go.jp; 4 General Education Center, Okinawa Prefectural University of Arts, 1–4 Shuri Tounokura, Naha, Okinawa 903-8602, Japan; fujitayo@okigei.ac.jp; 5 Radioisotope Center, The University of Tokyo, 2-11-16 Yayoi, Bunkyo-ku, Tokyo 113-0032, Japan; akimitsu@ric.u-tokyo.ac.jp; 6 Department of Microbiology, Faculty of Medicine, Graduate School of Interdisciplinary Research, University of Yamanashi, 1110 Shimokato, Chuo-shi, Yamanashi 409-3898, Japan; atsuyay@yamanashi.ac.jp (A.Y.); kmoriishi@yamanashi.ac.jp (K.M.); 7 Environmental Measurement Research Institute, National Institute of Advanced Industrial Science and Technology, 16-1 Onogawa, Tsukuba, Ibaraki 305-8569, Japan; h.tani@aist.go.jp; 8 Department of Pharmaceutical Sciences, Toho University, 2-2-1, Miyama, Funabashi-shi, Chiba 274-8510, Japan; captain.resonance@gmail.com; 9 Institute of Medical Chemistry, Hoshi University, 2-4-41 Ebara, Shinagawa-ku, Tokyo 142-8501, Japan; tsubuki@hoshi.ac.jp

**Keywords:** hepatitis C virus (HCV), NS3 helicase, crinoid, aromatic sulfates

## Abstract

Bioassay-guided separation of a lipophilic extract of the crinoid *Alloeocomatella polycladia*, inhibiting the activity of HCV NS3 helicase, yielded two groups of molecules: cholesterol sulfate and four new aromatic sulfates **1**–**4**. The structures of the aromatics were elucidated by spectroscopic analysis in addition to theoretical studies. The aromatic sulfates **1**–**4** showed moderate inhibition against NS3 helicase with IC_50_ values of 71, 95, 7, and 5 μM, respectively.

## 1. Introduction

Marine organisms have been attractive targets of novel drug discovery for decades [1]. As a result of this work, several drugs based on natural products and derivatives have been approved for clinical treatments. Among them, an analgesic drug ziconotide as well as antitumor drugs trabectedin and plitidepsin retain exactly the same chemical structures as in marine organisms, while antitumor agents cytarabine, eribulin, and brentuximab vedotin are based on marine natural products including a conjugate with an antibody [2]. The USFDA has approved seven drugs derived from marine organisms so far, however, this success rate, out of 28,000 registered marine products, can be taken favorably relative to terrestrial products [3].

In our campaign to discover molecules to interrupt the enzymes of hepatitis C virus, we previously reported the possibility of coral reef organisms as a source for new antivirals [4]. Among the extracts prepared from 61 marine organisms, we found that a lipophilic extract of the feather star *Alloeocomatella polycladia* exhibited inhibition with an IC_50_ of 11.7 μg/mL against hepatitis C virus (HCV) NS3 helicase. Bioassay-guided fractionation of the extract led to the isolation of two groups of molecules. One is cholesterol sulfate, and we previously reported its mode of inhibition with an IC_50_ value of 1.7 ± 0.2 μM against NS3 helicase by using an authentic molecule [5]. The other molecules were elucidated to be new aromatic sulfates **1**–**4**, and their structures and biological profiles are the subject of this manuscript.

## 2. Results and Discussion

**Figure 1:**
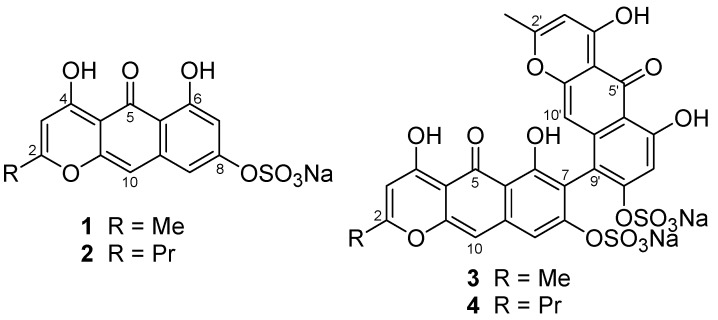
Structures of Compounds **1**–**4**.

From the first collection of the crinoid, we isolated Compounds **1**–**4** by chromatography (Figure 1). To supply an additional amount of Compound **1** for further characterization, we collected a second specimen. 

**Figure 2:**
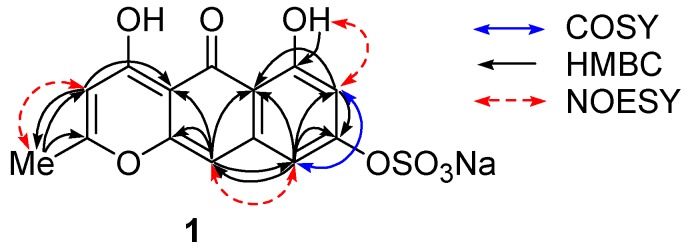
Representative 2D NMR correlations in Compound **1**.

The molecular formula, C_14_H_9_O_8_SNa, of Compound **1** was determined by observing a molecular-related ion at *m/z* 337.00237 [M − Na]^−^ in negative HRESIMS. A fragment ion at *m/z* 257.04609 [M − SO_3_Na]^−^ and an IR absorption at 1238 cm^−1^ supported the presence of a sulfate group, while analysis with atomic absorption confirmed it as a sodium salt. Of 14 carbon signals in the ^13^C NMR spectrum, 12 olefinic signals together with 10 degrees of unsaturation suggested the nature of Compound **1** as an aromatic polyketide, as reported for other crinoid metabolites [6]. The ^1^H NMR spectrum in DMSO-*d*_6_ revealed the presence of an aromatic methyl at δ 2.31 as well as four aromatic signals consisting of a pair of *m*-coupled protons at δ 6.33 and 6.79, and of two singlets at δ 5.93 and 6.56. These protons showed HMBC correlations as follows: H-7 (δ 6.33)/C-5a,8; H-9 (δ 6.79)/C-5a,7,8,10; H-3 (δ 5.93)/C-2,4a,5,11; H-10 (δ 6.56)/C-4a,5,5a,9,10a; Me-11 (δ 2.31)/C-2,3 (Figure 2, Table 1). A downfield-shifted phenolic proton at δ 14.30, showing HMBC correlations to C-5a, 6, and 7, was assigned to OH-6 and was expected to be involved in an internal hydrogen bond with a neighbor carbonyl group at δ 180.3 (C-5). NOESY cross peaks between Me-11/H-3 and OH-6/H-7 indicated their *ortho* relations, and the one between H-9/H-10 indicated *peri* position (Figure 2).

**Figure 3:**
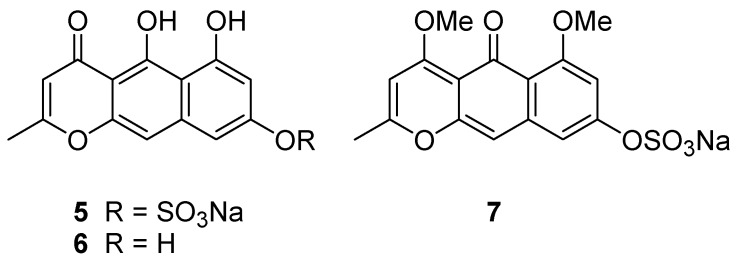
Structures of Compounds **5**–**7**.

Taking all the above information together, two tautomeric structures, **1** and **5**, are the candidate structures (Figure 3). Comparison of NMR data for nor-rubrofusarin (**6**) [7], a desulfated molecule of **5**, with that of Compound **1**, suggested they are neither identical to C-8, nor to the remaining portion. However, it was not clear enough to conclude one of the candidates is Compound **1**.

**Figure 4:**
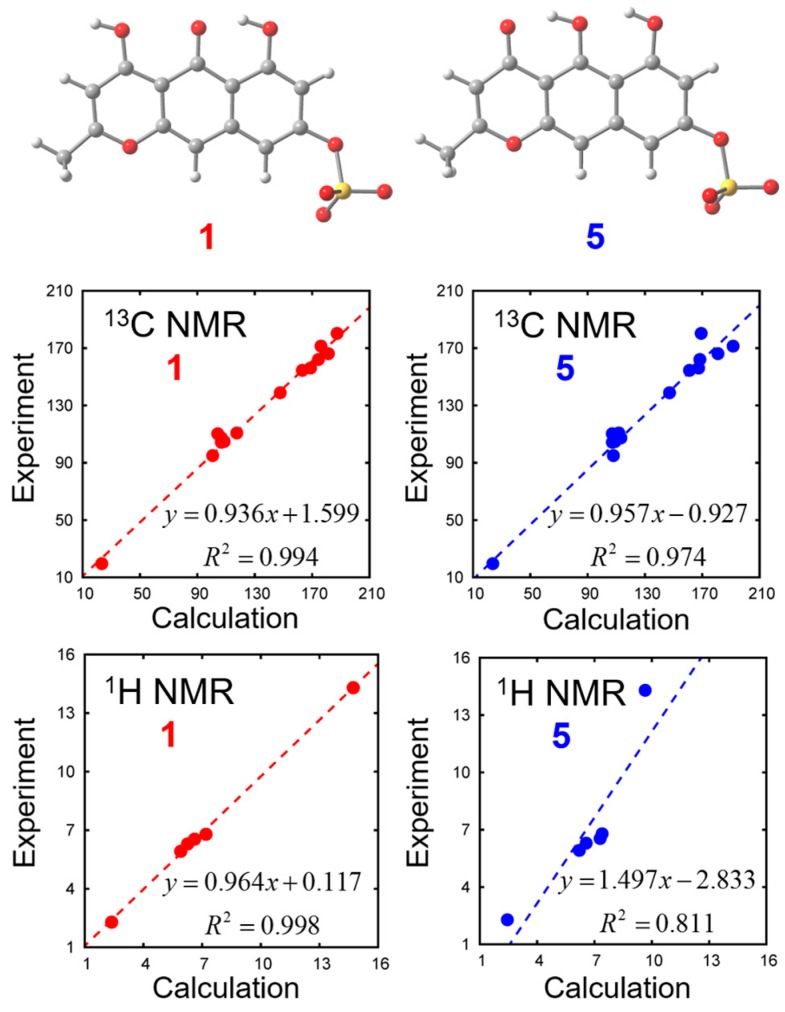
Optimized geometries and comparison of NMR data for Compounds **1** and **5**.

In order to distinguish the two tautomeric structures **1** and **5**, we calculated their chemical shifts with density functional theory (DFT) calculations. Solvent effects are incorporated with the polarizable continuum model (PCM). Optimized geometries and chemical shifts calculated at the PCM(DMSO)-B3LYP/6-311++G(d,p) level are summarized in Figure 4 and Table 2. We found that experimental NMR data is closer to the calculated values of Compound **1** than to Compound **5**.

Further confirmation was done with chemical derivatization. By treating Compound **1** with TMS-CHN_2_, we obtained dimethyl derivative **7** (*m/z* 365.03333 [M − Na]^−^), showing two methoxy groups at δ 3.80 and 3.91. Since the methoxy signal at δ 3.80 showed nuclear Overhauser effect (NOE) to the proton at δ 6.79 (H-7), it was confirmed to be at C-6. Another methoxy signal at δ 3.91 showed NOE to the proton at δ 5.99 (H-3), but not to the methoxy at δ 3.80. Therefore, the structure was concluded as shown in **1**.

Compound **2**, a yellow solid, was found to have a molecular formula C_16_H_13_O_8_SNa by observing a molecular-related ion at *m/z* 365.03317 [M − Na]^−^ in the negative HRESIMS. A sulfate group was recognized with a desulfated fragment ion at *m/z* 285.07650 [M − SO_3_Na]^−^ and the IR absorption at 1229 cm^−1^ as in Compound **1**. Since aromatic signals in the ^1^H NMR spectrum (δ 5.94 s, 6.48 d, 6.69 s, 6.96 d in MeOH-*d*_4_) of **2** were almost the same as those of **1** (δ 5.94 s, 6.48 d, 6.67 s, 6.96 d in MeOH-*d*_4_) as well as the same UV absorption maxima as **1**, Compound **2** has the same ring system as **1**. Additional two methylene units are incorporated into a propyl chain (δ 1.03 t, 1.77 sext, 2.57 t) instead of a methyl in Compound **1**, and its position was confirmed by HMBC cross peaks at H-11/C-2,3 (Table 1). Compound **2** was concluded to be a propyl analog of **1**.

The molecular formula of Compound **3**, C_28_H_16_Na_2_O_16_S_2_, was determined by observing a molecular-related ion at *m/z* 694.97765 [M − Na]^−^ and a desulfated ion at *m/z* 593.04142 [M + H − SO_3_Na_2_]^−^. The presence of sulfate groups was also confirmed by the IR spectrum (1240 cm^−1^). Since the molecular formula of **3** is almost the double of Compound **1** with two fewer hydrogen atoms and a monomeric fragment ion at *m/z* 335.9945 [C_14_H_8_O_8_S]^−^ was observed, the dimeric nature of Compound **3** was apparent. The ^1^H NMR data in DMSO-*d*_6_ showed two sets of signals: two methyl signals at δ 2.18 and 2.34, six aromatic singlets at δ 5.85, 5.92, 6.06, 6.53, 7.11, and 7.34, and two hydrogen-bonded phenolic protons at δ 14.20 and 14.40, indicating that Compound **3** is an asymmetric dimer. After analysis by 2D NMR, nearly the same chemical shifts were seen for H-10 (Δδ + 0.01), H-3 (0.00), H-11 (−0.05), H-3′ (−0.07), and H-11′ (+0.11) between Compounds **1** and **3**, whereas larger chemical shift differences were observed for H-9 (Δδ + 0.55), H-7′ (+0.81), and H-10′ (−0.48), probably caused by deshielding/shielding effect of another aromatic ring in **3**. In addition, the following HMBC correlations point to the two units being connected at C-7 and C-9′ (Table 3): OH-6 (δ 14.20)/C-5a,6,7 and H-9 (δ 7.34)/C-5a,7,8,10 for one of the monomeric units and OH-6′ (δ 14.40)/C-5a′,6′,7′ and H-7′ (δ 7.11)/C-5a′,6′,8′,9′ for another unit.

Since the UV absorption maxima are similar between Compounds **1** and **3**, two aromatic units do not exist in the same plane due to the bulkiness of sulfate and hydroxyl groups. In fact, the specific rotation value, [α]_D_ −94, supports the presence of axial chirality. The ECD spectrum showed a negative Cotton effect at 291 nm (Δε −74.5) and at 270 nm (+124), indicating an anticlockwise arrangement of the two aromartic moieties.

Compound **4** was shown to have a molecular formula C_30_H_20_Na_2_O_16_S_2_ with a molecular-related ion at *m/z* 723.00759 [M − Na]^−^ in ESIMS. Analogous to Compound **3**, similar chemical shift differences were observed for H-9 (Δδ +0.51), H-7′ (+0.65), and H-10′ (−0.26), indicating the connection between C-7 and C-9′ with all NMR signal assignments (Table 3). The structural difference between **3** and **4** lies at one of the alkyl chains—*n*-propyl group (δ 2.59 t, 1.80 sext, 1.06 t, H-11~13)—in Compound **4** instead of a methyl (δ 2.34 s, H-11) in Compound **3**. The propyl group was shown to be attached to C-2 after observing HMBC correlations H-3/C-4a,5,11, H-10/C-4a,5,5a, and H-9/C-5a. As in the case of Compound **3**, Compound **4** showed similar values for specific rotation, [α]_D_ −87, and a negative Cotton effect at 291 nm (Δε −69.8) and 270 nm (+122), suggesting the same arrangement of two aromatic moieties as that of **3**.

**Figure 5:**
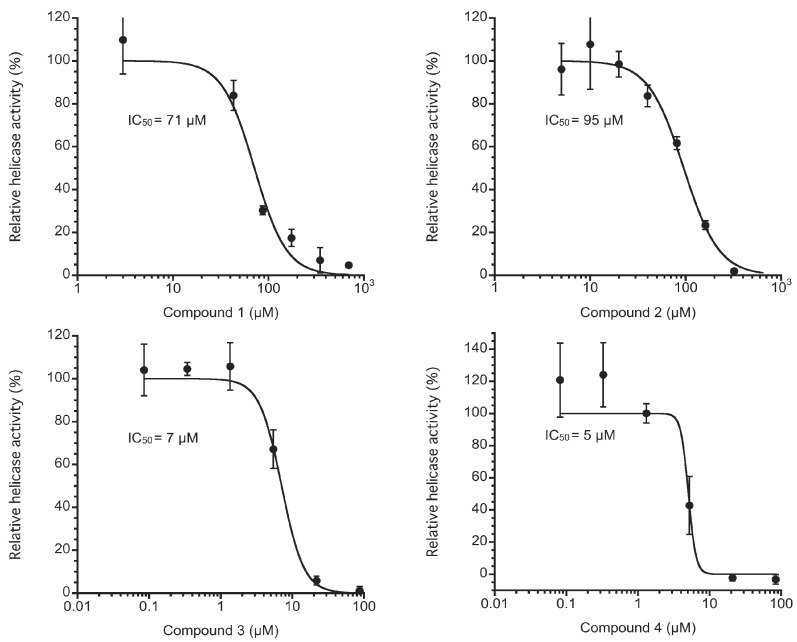
Inhibition curves of Compounds **1**–**4** generated using the fluorescence helicase assay.

For enzymatic inhibition, we used the fluorescence helicase assay based on fluorescence resonance energy transfer (FRET) [8] to examine NS3 helicase inhibition by Compounds **1**–**4**. Because Compounds **1**–**4** showed a wide range of absorption wavelengths up to ~500 nm, we used a double-stranded RNA substrate (dsRNA) labeled with Alexa Fluor 700 and Black Hole Quencher 3 to avoid interference due to absorption. The measured values in triplicate for each concentration of Compounds **1**–**4** were averaged and divided by the mean value from the negative control sample that contains DMSO instead of Compounds **1**–**4** to show the NS3 helicase activity as a percentage. Compounds **1**–**4** inhibited NS3 helicase with IC_50_ values of 71, 95, 7, and 5 μM, respectively, as shown in Figure 5. Because the chemical structures of Compounds **1**–**4** appear similar to those of hydroxyanthraquinones, this result may be consistent with our previous results in which hydroxyanthraquinones were found to inhibit NS3 helicase [8]. Interestingly, Compounds **3** and **4**, which have a dimeric form consisting of Compound **1** and a form consisting of Compounds **1** and **2**, respectively, exerted stronger inhibition than Compounds **1** and **2** did. This result may also be consistent with our previous results in which a dimeric form of hydroxyanthraquinones was found to exert stronger inhibition than a monomeric form did, as previously described [8].

Further, we investigated the effect of Compounds **2** and **3** on both viral replication and cell growth in the HCV subgenomic replicon cell line Huh7/Rep-Feo (genotype 1b, N strain) [9]. Compounds **2** and **3** dose-dependently suppressed HCV replication and exhibited the EC_50_ (50% effective concentration) values of 57 and 67 μM, respectively. The CC_50_ (50% cytotoxicity concentration) values of Compounds **2** and **3** were 52 and 70 μM, respectively. These results suggest that the NS3 helicase inhibition was not well correlated with the anti-HCV activity of Compounds **2** and **3**, indicating that the compounds were not well delivered into cytoplasm of the Huh7/Rep-Feo cells. This was likely due to its low membrane permeability caused by the negatively charged sulfate group within the molecule in aqueous solution.

## 3. Materials and Methods

### 3.1. General Experimental Procedures

The 1D and 2D NMR spectra were recorded on a Bruker AVANCE III 500 spectrometer (Bruker, Rheinstetten, Germany). The mass spectra were measured on a JEOL JMS-T100LP mass spectrometer with ESI as an ionization source. The FTIR spectra were measured using a JASCO FT/IR-6100 UV spectra (Japan Spectroscopic Corporation, Tokyo, Japan) were taken on a Hitachi U-2001 spectrophotometer (Hitachi High-Tech Corporation, Tokyo, Japan). Optical rotation was measured on a Jasco P-1010 polarimeter, and ECD spectra were taken on a Jasco J-820 instrument. The presence of sodium was measured on a Hitachi Z-2010 atomic absorption spectrophotometer. High-performance liquid chromatography (HPLC) was performed on a Hitachi L-6000 pump equipped with a Shodex SE-101 RI monitor (Showa Denko, Tokyo, Japan) and a Hitachi L-4000 UV detector using Imtakt Cadenza CD-C18 (10 mm × 250 mm), or Nacalai Cosmosil 5C_18_-ARII (4.6 mm × 250 mm), or YMC Pack ODS-AQ (4.6 mm × 100 mm) column. Nacalai Cosmosil 75C_18_-PREP reversed phase gel was used for vacuum flash chromatography. All solvents used were reagent grade.

### 3.2. Animal Material

Specimens of the crinoid *Alloeocomatella polycladia* were collected by hand using scuba at 3–10 m depth at Mizugama, Okinawa, June 2009, and the material was kept frozen until extraction. To supply additional material, another collection of the crinoids was done at the same place in June 2016. The specimen was identified by one of us (YF).

### 3.3. Extraction and Isolation

The first specimen of the crinoid *A. polycladia* (179 g, wet) was broken into small pieces and soaked in acetone (1 L × 3) for 20 h, then in MeOH (1 L × 1) for 6 h. The acetone and MeOH solutions were combined and concentrated, and the residual material was partitioned between EtOAc and H_2_O. The H_2_O layer was dried to yield a residue (4.6 g), which was subjected to ODS flash chromatography with stepwise gradient elution using 0, 25, 50, 75, and 100% MeOH in H_2_O to yield five fractions. A portion (75.4 mg) of the third fraction was applied to a Sephadex LH20 (GE Healthcare Life Sciences, Pittsburgh, USA) column followed by reversed phase HPLC (H_2_O-MeCN, 4-1) to yield Compounds **1** (3.0 mg) and **3** (8.2 mg). Most (70.0 mg) of the fourth fraction (81.1 mg) from the flash chromatography was separated with Sephadex LH20 (MeOH) and ODS HPLC (H_2_O-MeCN, 3-1) to yield Compounds **2** (1.6 mg) and **4** (5.5 mg).

The second specimen, 282 g (wet), was treated in a similar way as previous work. After partition between EtOAc and H_2_O, the H_2_O layer was dried to yield a residue (8.92 g). This residue was subjected to ODS flash chromatography with stepwise gradient elution in the same manner to yield five fractions. The MeOH soluble portion (557.9 mg) of the first fraction (8.42 g) was applied to a Sephadex LH20 column (MeOH) to yield six subfractions. The fourth subfraction (187.4 mg) was introduced to reversed phase gradient HPLC (5%–50% of MeCN in H_2_O, totally 30 min) to yield Compound **1** (9.8 mg). Using the same procedure, 259.9 mg of the second fraction from the flash chromatography was separated to yield Compounds **3** (7.2 mg) and **4** (6.3 mg).

Compound **1**. yellow solid; UV (MeOH) λ_max_ nm (log ε) 440 (3.2), 339 (3.6), 277 (4.4), 221 (4.1); IR (KBr) 3421, 1640, 1238 cm^−1^; HRESIMS *m/z* 337.00237 ([M − Na]^−^, calcd. for C_14_H_9_O_8_S 337.00181, Δ +0.55 mmu), 257.04609 ([M − SO_3_Na]^−^, calcd. for C_14_H_9_O_5_, 257.04500, Δ +1.09 mmu); ^1^H and ^13^C NMR (MeOH-*d*_4_ and DMSO-*d*_6_) see Table 1. AAS: C_Na_ = 0.29 ppm. 

Compound **2**. yellow solid; UV (MeOH) λ_max_ nm (log ε) 438 (3.2), 339 (3.7), 277 (4.3), 220 (4.1); IR (KBr) 3402, 1641, 1238 cm^−1^; HRESIMS *m/z* 365.03317 ([M − Na]^−^, calcd. for C_16_H_13_O_8_S 365.03311, Δ +0.06 mmu), *m/z* 285.07650 (calcd. for C_16_H_13_O_5_ 285.07685, Δ −0.35 mmu); ^1^H and ^13^C NMR (MeOH-*d*_4_) see Table 1.

Compound **3**. brown solid; [α]_D_ −94 (*c* 0.29, MeOH); UV (MeOH) λ_max_ nm (log ε) 445 (3.4), 343 (3.9), 278 (4.5), 222 (4.4); IR (KBr) 3420, 1643, 1240 cm^−1^; HRESIMS *m/z* 694.97765 ([M − Na]^−^, calcd. for C_28_H_16_NaO_16_S_2_ 694.97830, Δ −0.65 mmu), *m/z* 593.04142 ([M + H − SO_3_Na_2_]^−^, calcd. for C_28_H_17_O_13_S, 593.03954, Δ −0.64 mmu), *m/z* 335.9945; ^1^H and ^13^C NMR (DMSO-*d*_6_) see Table 3; ECD (MeOH) 346.0 (Δε −21.3), 291.2 (−74.5) and 270.4 nm (+124).

Compound **4**. brown solid; [α]_D_ −87 (*c* 0.27, MeOH); UV (MeOH) λ_max_ nm (log ε) 447 (3.5), 343 (4.1), 279 (4.5), 223 (4.4); IR (KBr) 3402, 1641, 1238 cm^−1^; HRESIMS *m/z* 723.00759 ([M − Na]^−^, calcd. for C_30_H_20_NaO_16_S_2_ 723.00960, Δ −2.01 mmu); ^1^H and ^13^C NMR (MeOH-*d*_4_) see Table 3; ECD (MeOH) 345.8 (Δε −21.1), 291.2 (−69.8) and 270.4 nm (+122).

Methylation of **1**. A solution of Compound **1** (1.1 mg) in MeOH (350 μL) was treated with an excess of TMS-CHN_2_, and the mixture was allowed to stand for 24 h. The product was purified by preparative TLC (silica, hexane-EtOAc, 5-1) to yield Compound **7** (0.5 mg, 42%).

Compound **7**. yellow solid; HRESIMS *m/z* 365.03333 ([M − Na]^−^, calcd for C_16_H_13_O_8_S 365.03311, Δ +0.22 mmu), 285.07579 ([M − SO_3_Na]^−^, calcd for C_16_H_13_O_5_, 285.07630, Δ −0.51 mmu); ^1^H NMR (DMSO-*d*_6_) δ 7.61 (s, H-10), 7.30 (d, *J* = 2. 1 Hz, H-9), 6.79 (d, *J* = 2.1 Hz, H-7), 5.99 (s, H-3), 3.91 (s, 6-OMe), 3.80 (s, 4-OMe), 2.32 (s, H-11).

### 3.4. DFT Calculation for Compounds ***1*** and ***5***

We calculated the NMR chemical shifts of Compounds **1** and **5** by using the Gauge-Independent Atomic Orbital (GIAO) method [10]. The B3LYP density functional was employed with 6-311++G(d,p) basis set. The solvation effects of DMSO were included using the integral equation formalism polarizable continuum model (IEFPCM) [11]. We found 8 and 6 conformations in total for Compounds **1** and **5** with respect to the orientations of 1 sulfate and 2 hydroxy groups. Among these conformations, the chemical shifts most close to the experimental values are shown in Figure 4 and Table 2. All calculations were carried out with the Gaussian 09 program [12] at the Research Center for Computational Science, Okazaki, Japan.

### 3.5. HCV NS3 Helicase Assay

The fluorescence helicase assay based on FRET was conducted as we previously described [8], except that we used a DNA capture strand rather than an RNA capture strand for which the sequence was complementary to that of the quencher strand. HCV NS3 protein with helicase activity was expressed and purified as previously described [13]. In the assay, when NS3 helicase unwinds the dsRNA substrate, the DNA capture strand binds to the unwound single-stranded RNA (ssRNA) substrate labeled with Black Hole Quencher 3 and prevents the ssRNA substrate from reannealing to the complementary ssRNA substrate labeled with Alexa Fluor 700. This results in increased fluorescence intensity of Alexa Fluor 700 apart from Black Hole Quencher 3. Thus, the NS3 helicase activity can be monitored by the real-time measurement of the fluorescence intensity of Alexa Fluor 700 through 30 min incubation at 37 °C by using a SpectraMax Gemini XS microplate reader (Molecular Devices, Sunnyvale, CA, USA). The reaction mixture contained 25 mM MOPS-NaOH (pH 6.5), 3 mM MgCl_2_, 2 mM dithiothreitol, 4 U RNasin (Promega, Madison, WI, USA), 50 nM dsRNA substrate, 400 nM DNA capture strand, 5 mM ATP, a serial dilution of Compounds **1**–**4** dissolved in DMSO, and 240 nM NS3 helicase in a total reaction volume of 20 μL. The NS3 helicase activity was calculated as the initial reaction velocity relative to that of the control (in the absence of Compounds **1**–**4** but in the presence of DMSO). The IC_50_ value was calculated using KaleidaGraph (Synergy Software, Reading, PA, USA) by fitting plots of relative helicase activity (%) vs. [*I*] using Equation (1):

(1) $$\%\;\mathrm{Activity}\;=\;\frac{100}{1\;+\;{\left(\left[I\right]/{\mathrm{IC}}_{50}\right)}^{h}}$$

where *h* is the Hill coefficient, and [*I*] is the inhibitor concentration.

### 3.6. HCV Replicon Assay

The Huh7/Rep-Feo cell line harboring the subgenomic replicon RNA of the N strain (genotype 1b) was seeded at 2 × 10^4^ cells per well in a 48-well plate and incubated at 37 °C for 24 h. A serial dilution of Compounds **2** and **3** was added to each well of the plate. The treated cells were harvested 72 h post-treatment and lysed in cell culture lysis reagent (Promega, Madison, WI, USA). Luciferase activity in the harvested cells was estimated with a luciferase assay system (Promega). The resulting luminescence was detected by a Luminescencer-JNR AB-2100 (ATTO, Tokyo, Japan). The anti-HCV activity was calculated as the intensity of the luminescence relative to that of the control (in the absence of Compounds **2** and **3** but in the presence of DMSO).

### 3.7. Cytotoxicity Assay

Huh7/Rep-Feo cells were seeded at a density of 1 × 10^4^ cells per well in a 96-well plate and then incubated at 37 °C for 24 h. Compounds **2** and **3** were added to the culture medium to yield various concentrations and were then harvested 72 h post-treatment. Cell viability was determined by a dimethyl thiazolcarboxymethoxyphenyl sulfophenyltetrazolium (MTS) assay using a CellTiter 96 aqueous one-solution cell proliferation assay kit (Promega), according to the manufacturer’s protocol.

## Figures and Tables

**Table 1:** ^1^H and ^13^C NMR data for Compounds **1** and **2**.

C#	1					2		
	^13^C	^1^H	HMBC	^13^C	^1^H	^13^C	^1^H	HMBC
	DMSO-*d*_6_			MeOH-*d*_4_		MeOH-*d*_4_		
2	166.4			168.7		171.8		
3	107.4	5.93 s	2, 4a, 11	108.5	5.94 s	108.1	5.94 s	2, 4a, 11
4	171.2			173.0		173.1		
4a	104.7			107.1		107.3		
5	180.3			183.6		183.6		
5a	110.7			113.4		113.4		
6	162.1	OH, 14.30 s	5a, 6, 7	163.8		163.8		
7	100.5	6.33 d, *J* = 2.1 Hz	5a, 8	102.1	6.48 d, *J* = 2.0 Hz	102.1	6.48 d, *J* = 2.2 Hz	5a, 6, 8, 9
8	156.1			156.6		156.6		
9	104.4	6.79 d, *J* = 2.1 Hz	5a, 7, 8, 10	107.1	6.96 d, *J* = 2.0 Hz	107.2	6.96 d, *J* = 2.2 Hz	5a, 7, 8, 10
9a	138.9			141.1		141.1		
10	95.1	6.56 s	4a, 5a, 9, 10a	97.9	6.67 s	97.9	6.69 s	5, 5a, 9, 9a, 10a
10a	154.6			156.5		156.4		
11	19.7	2.31 s	2, 3	20.2	2.31 s	36.8	2.57 t, *J* = 7.5 Hz	2, 3
12						21.3	1.77 sext, *J* = 7.5 Hz	2, 11
13						13.9	1.03 t, *J* = 7.4 Hz	11, 12

**Table 2:** Calculated NMR data for Compounds **1** and **5**.

C#	1		5	
	^13^C	^1^H	^13^C	^1^H
2	181.3		180.9	
3	106.7	5.91	113.2	6.21
4	176.1	(OH, 6.17) ^a^	191.5	
4a	108.6		109.1	
5	187.4		169.2	(OH, 15.38) ^a^
5a	117.4		111.7	
6	174.4	OH, 14.73	168.3	OH, 9.65
7	104.2	6.26	107.2	6.57
8	168.8		167.4	
9	106.7	7.20	107.3	7.40
9a	147.7		147.1	
10	100.6	6.61	108.0	7.30
10a	163.2		161.1	
11	23.4	2.37	23.8	2.44

^a^ These shifts are not observed experimentally.

**Table 3:** ^1^H and ^13^C NMR data for Compounds **3** and **4**.

C#	3			4		
	^13^C	^1^H	HMBC	^13^C	^1^H	HMBC
	DMSO-*d*_6_			MeOH-*d*_4_		
2	166.0			171.4		
3	107.4	5.92 s	2, 4, 4a, 11	108.0	5.93 s	2, 4a, 11
4	171.6			172.9		
4a	104.6			107.1		
5	180.3			183.5		
5a	110.3			113.1		
6	159.9	OH, 14.20 s	5a, 6, 7	161.8		
7	106.7			108.2		
8	155.6			155.9		
9	102.4	7.34 s	5a, 7, 8, 10	104.7	7.47 s	5a, 7, 8, 10
9a	137.9			140.5		
10	94.8	6.53 s	4a, 5a, 9, 9a, 10a	97.8	6.74 s	4a, 5, 5a, 9, 10a
10a	154.2			156.3		
11	19.7	2.34 s	2, 3	36.9	2.59 t, *J* = 7.4 Hz	2, 3, 12, 13
12				21.3	1.80 sext, *J* = 7.4 Hz	2, 11, 13
13				13.9	1.06 t, *J* = 7.4 Hz	11, 12
2′	165.9			168.6		
3′	107.0	5.85 s	2′, 4′, 4a′, 11′	108.2	5.86 s	2′, 4a′, 11′
4′	171.4			173.3		
4a′	104.1			106.8		
5′	180.3			183.6		
5a′	110.3			113.3		
6′	160.7	OH, 14.40 s	5a′, 6′, 7′	163.0		
7′	99.7	7.11 s	5a′, 6′, 8′, 9′	101.3	7.13 s	5a′, 6′, 8′, 9′
8′	154.7			154.9		
9′	107.4			111.0		
9a′	135.5			140.9		
10′	94.6	6.06 s	4a′, 5a′, 9′, 10a′	97.8	6.41 s	4a′, 5′, 5a′, 9′, 10a′
10a′	153.5			155.9		
11′	19.6	2.18 s	2′, 3′	20.2	2.19 s	2′, 3′

## Supplementary Materials

The spectral data of Compounds **1**–**4** are provided at www.mdpi.com/1660-3397/15/4/117/s1.
