# Supplementary material for: Four Aromatic Sulfates with an Inhibitory Effect against HCV NS3 Helicase from the Crinoid Alloeocomatella polycladia
Source: Mar Drugs. 2017 Apr 11;15(4):117. doi: 10.3390/md15040117 (PMC5408263; doi:10.3390/md15040117)
Supplement: Supplementary file 1 [file marinedrugs-15-00117-s001.pdf]

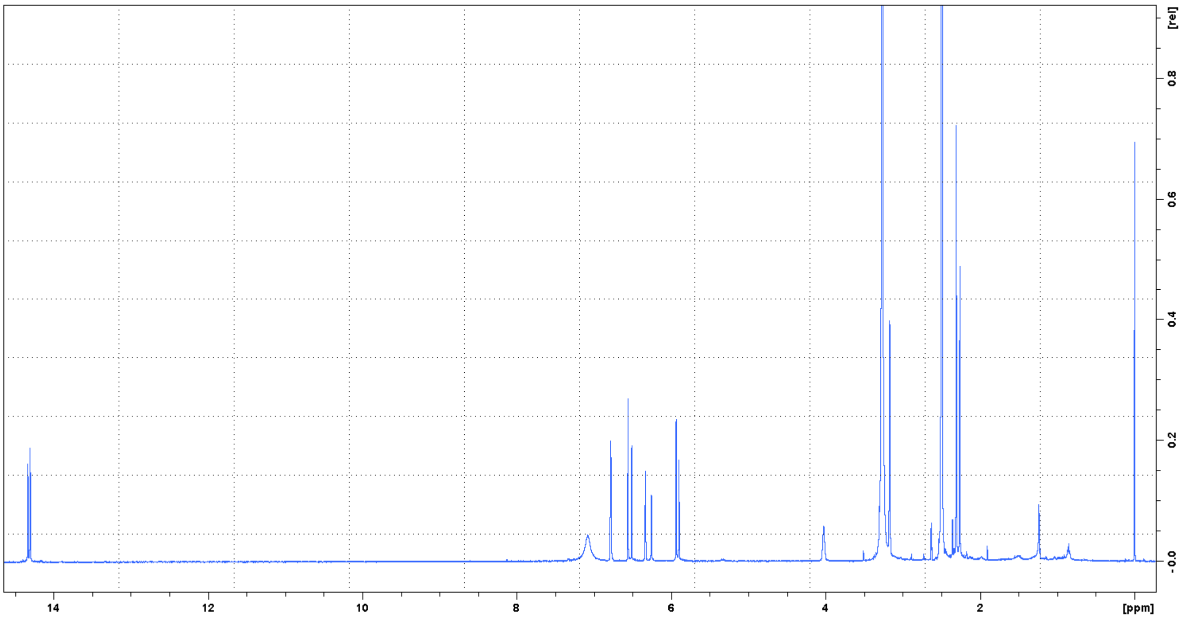

Figure 1. ^{1}H NMR spectrum of compound **1** in DMSO-d_6

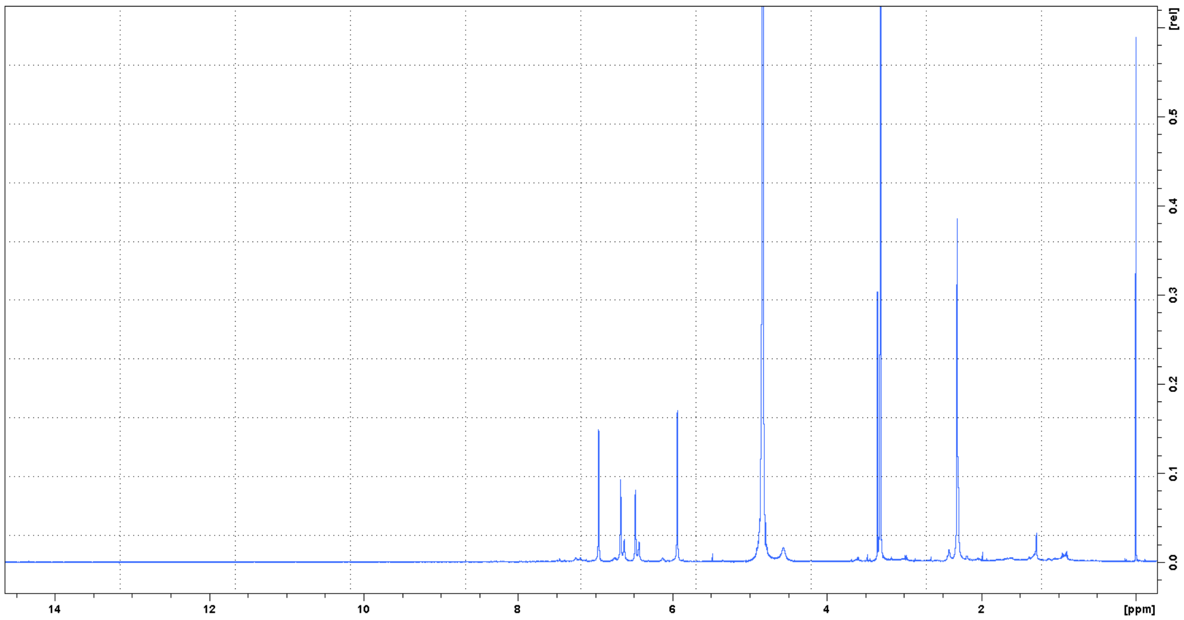

Figure 2. ^{1}H NMR spectrum of compound **1** in MeOH-d_4

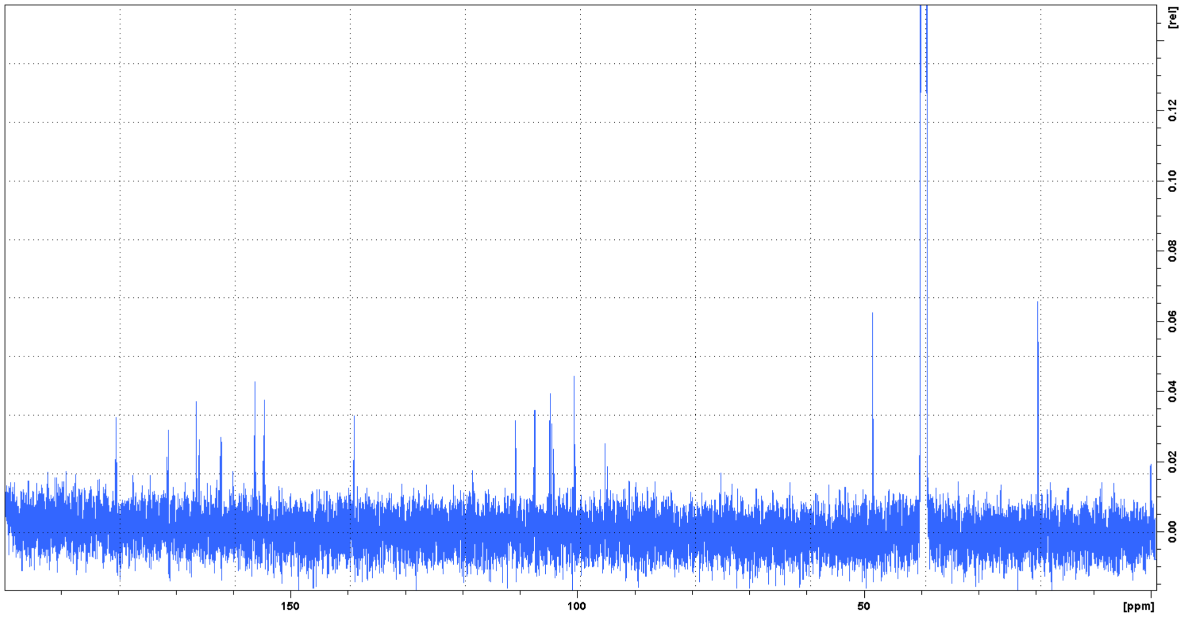

Figure 3. ^{13}C NMR spectrum of compound **1** in DMSO-d_6

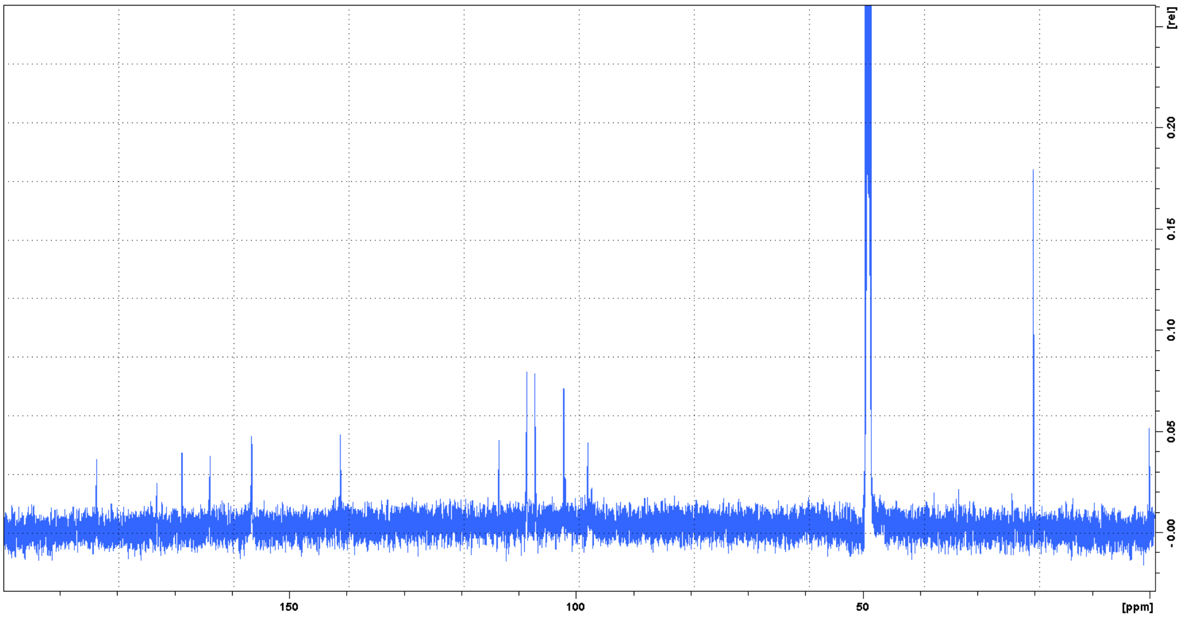

Figure 4. ^{13}C NMR spectrum of compound **1** in MeOH-d_4

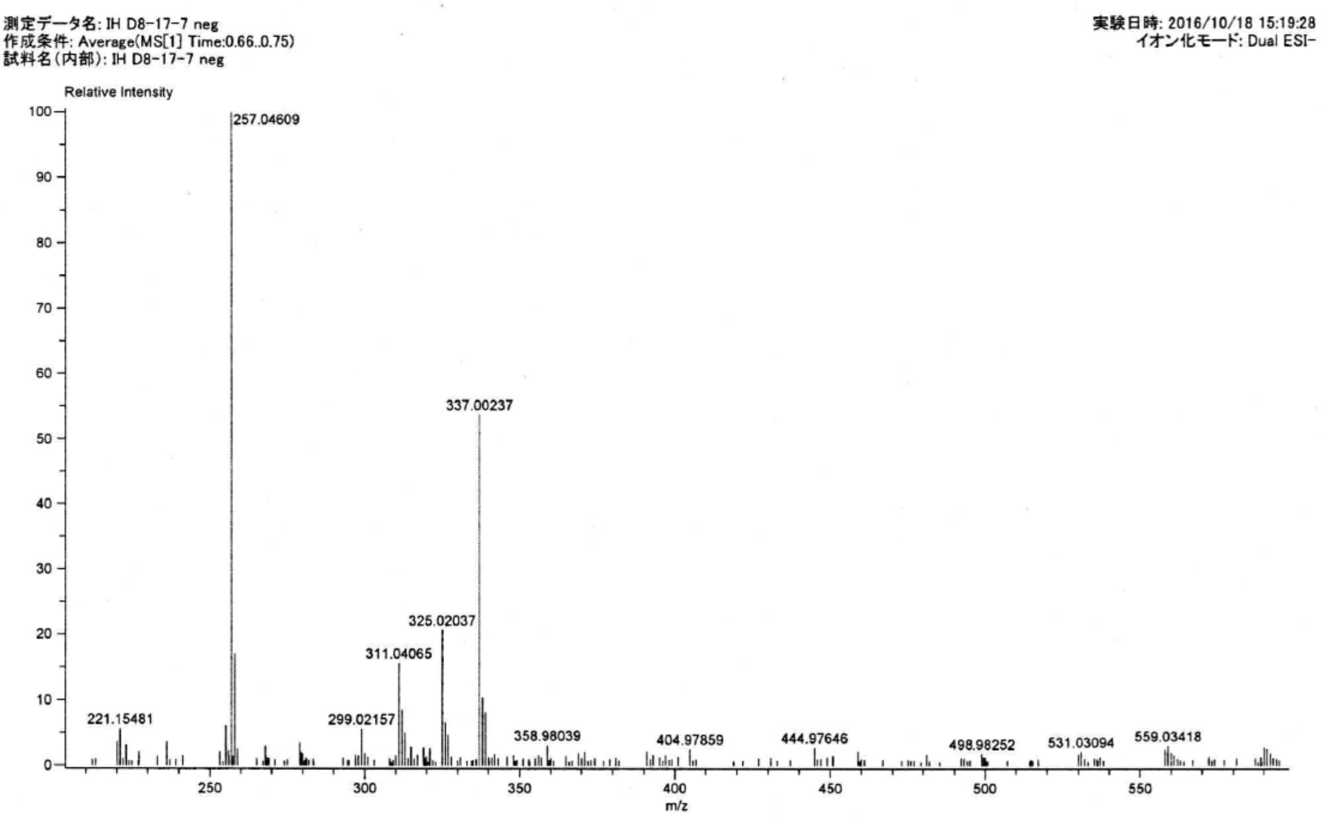

Figure 5.   ESIMS of compound **1**

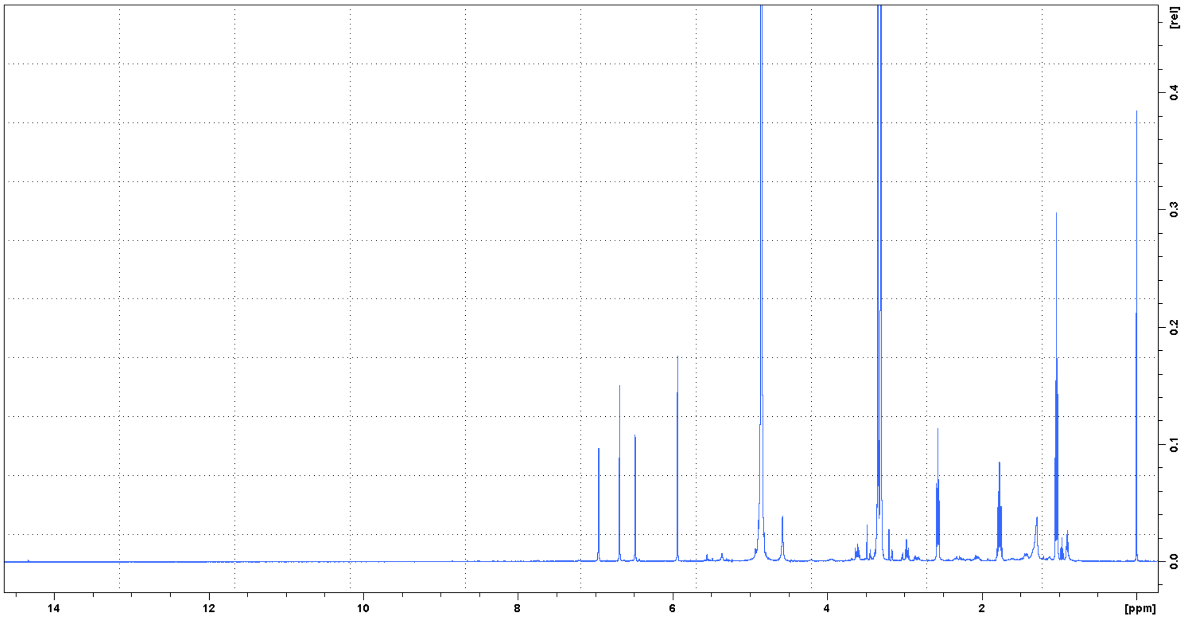

Figure 6. ^{1}H NMR spectrum of compound **2** in MeOH-d_4

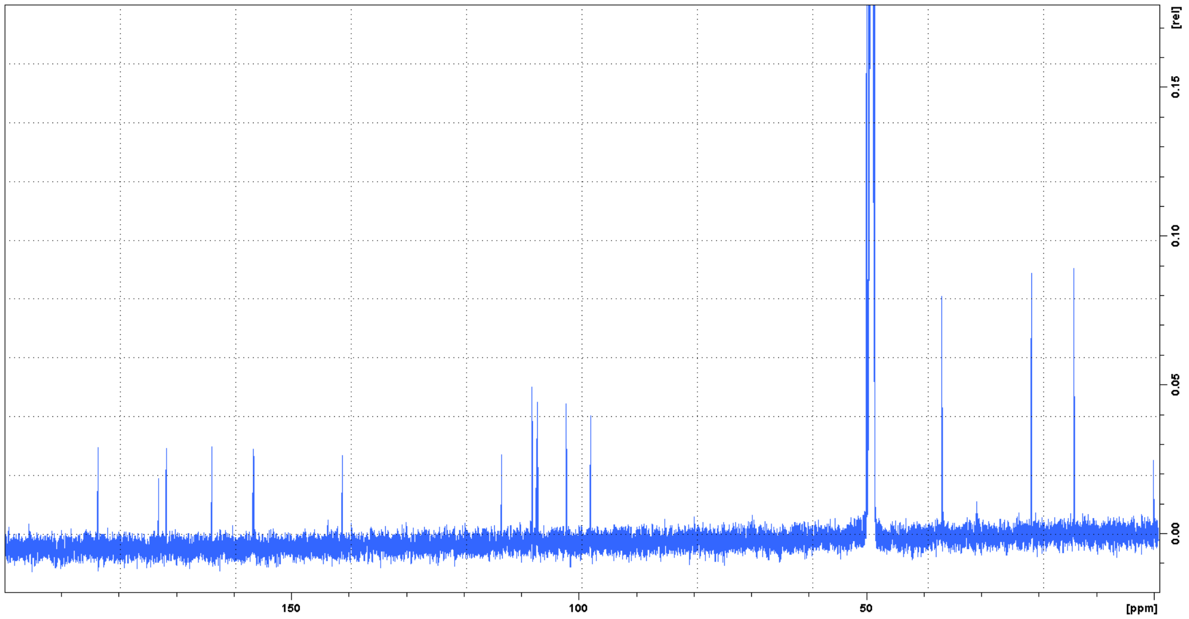

Figure 7. ^{13}C NMR spectrum of compound **2** in MeOH-d_4

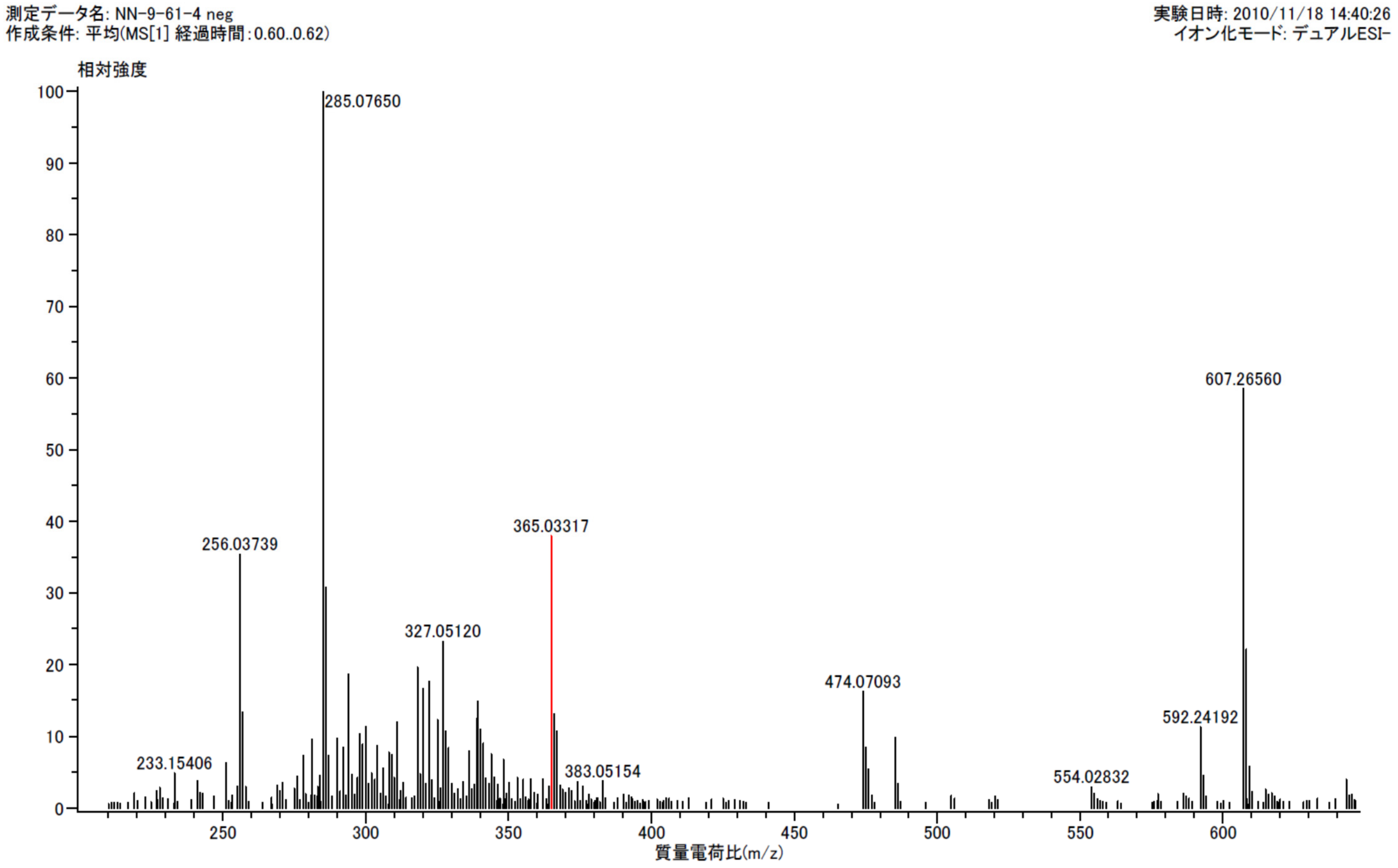

Figure 8. ESIMS of compound **2**

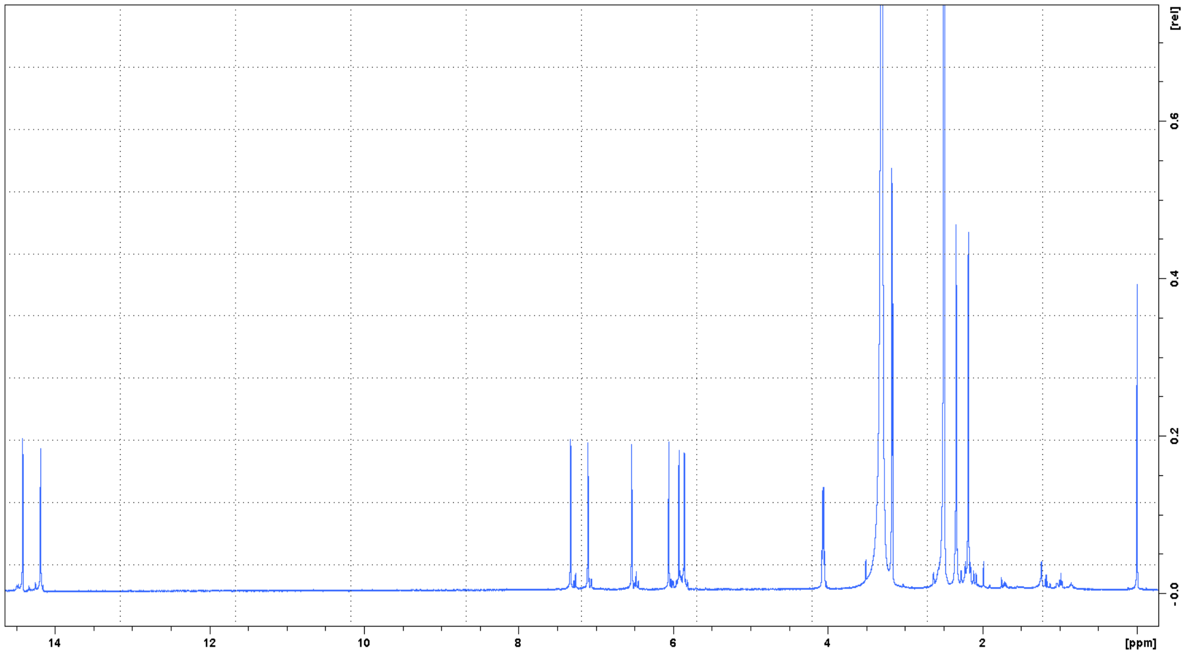

Figure 9. ^1H NMR spectrum of compound **3** in DMSO-d_6

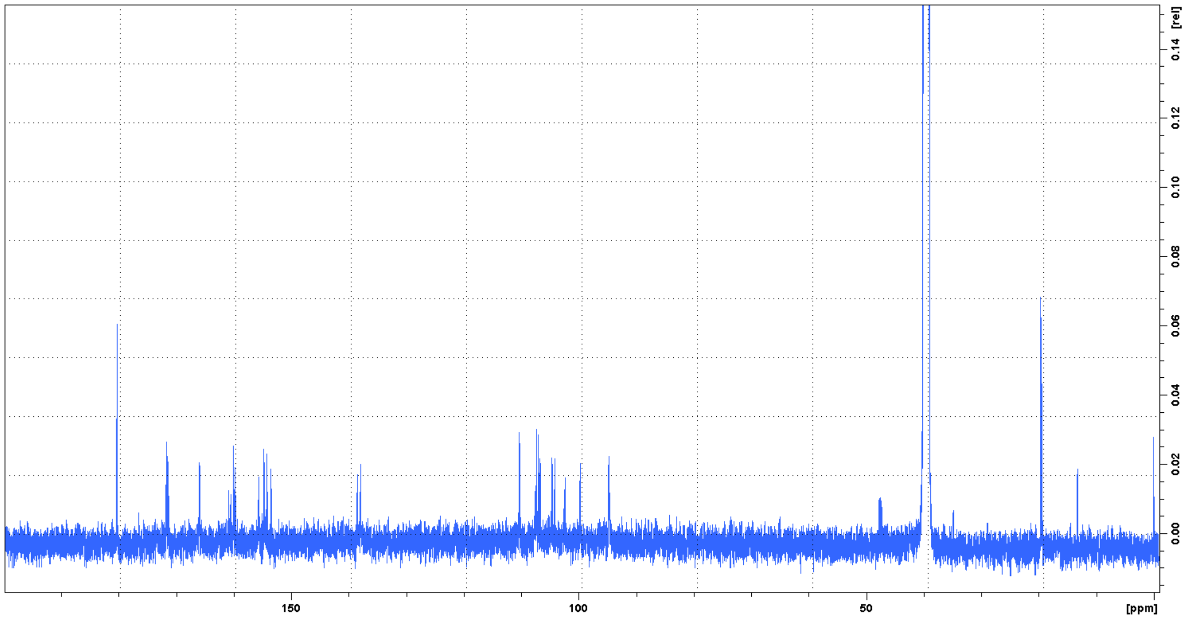

Figure 10. ^{13}C NMR spectrum of compound **3** in DMSO-d_6

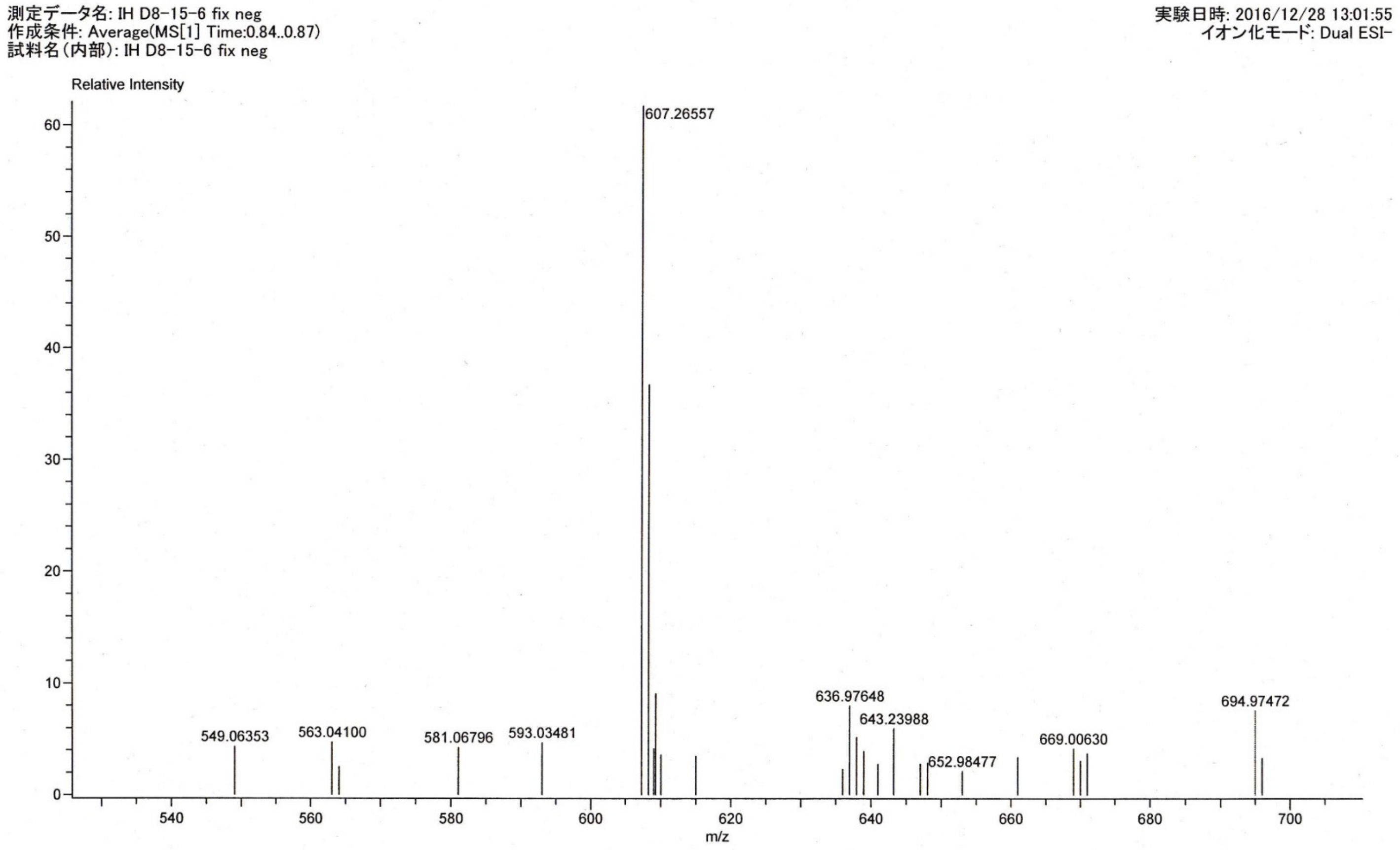

Figure 11. ESIMS of compound **3**

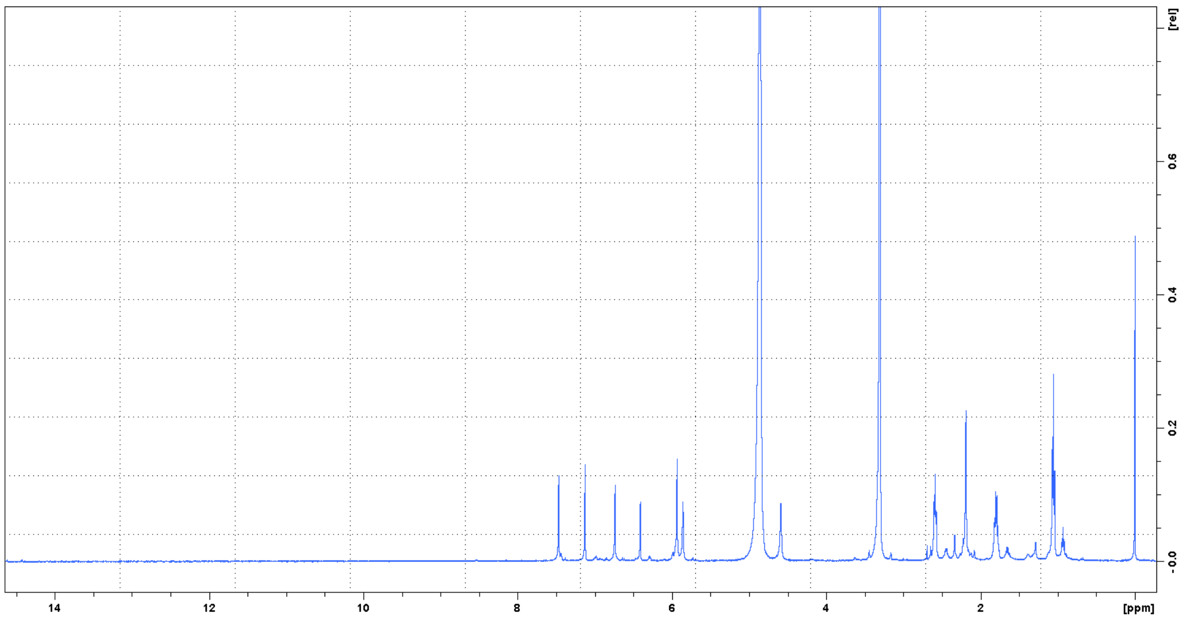

Figure 12. ^{1}H NMR spectrum of compound **4** in MeOH-d_4

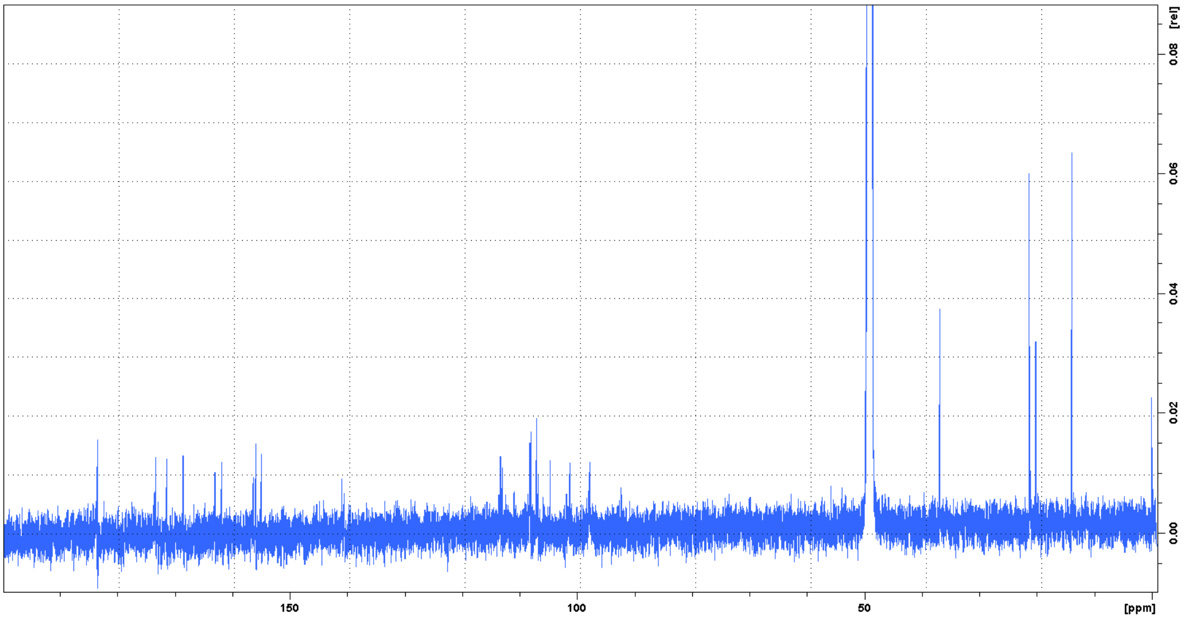

Figure 13. ^{13}C NMR spectrum of compound **4** in MeOH-d_4

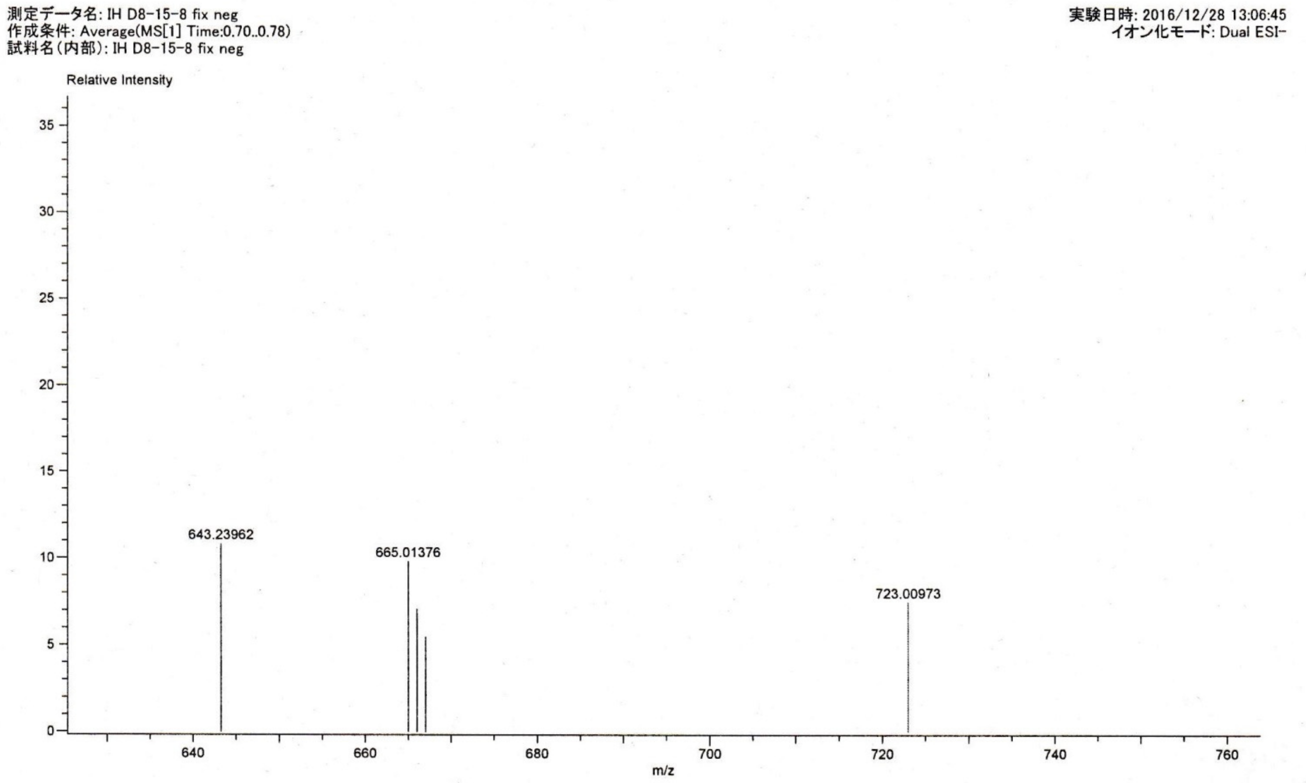

Figure 14. ESIMS of compound **4**

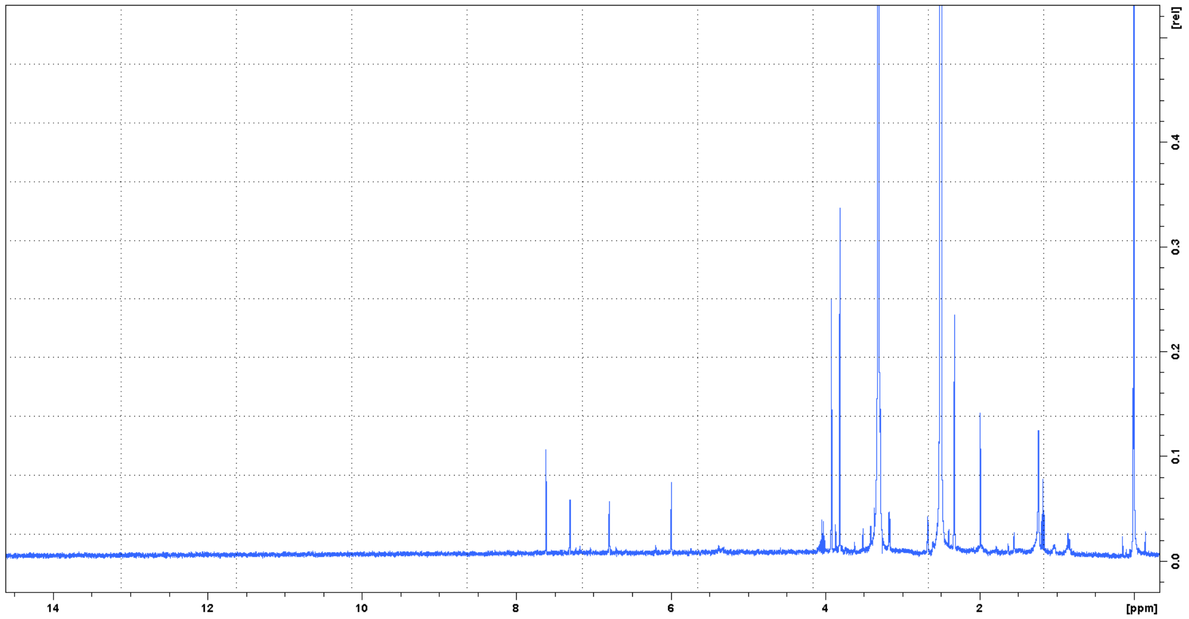

Figure 15. ^{1}H NMR spectrum of compound **7** in DMSO-d_6

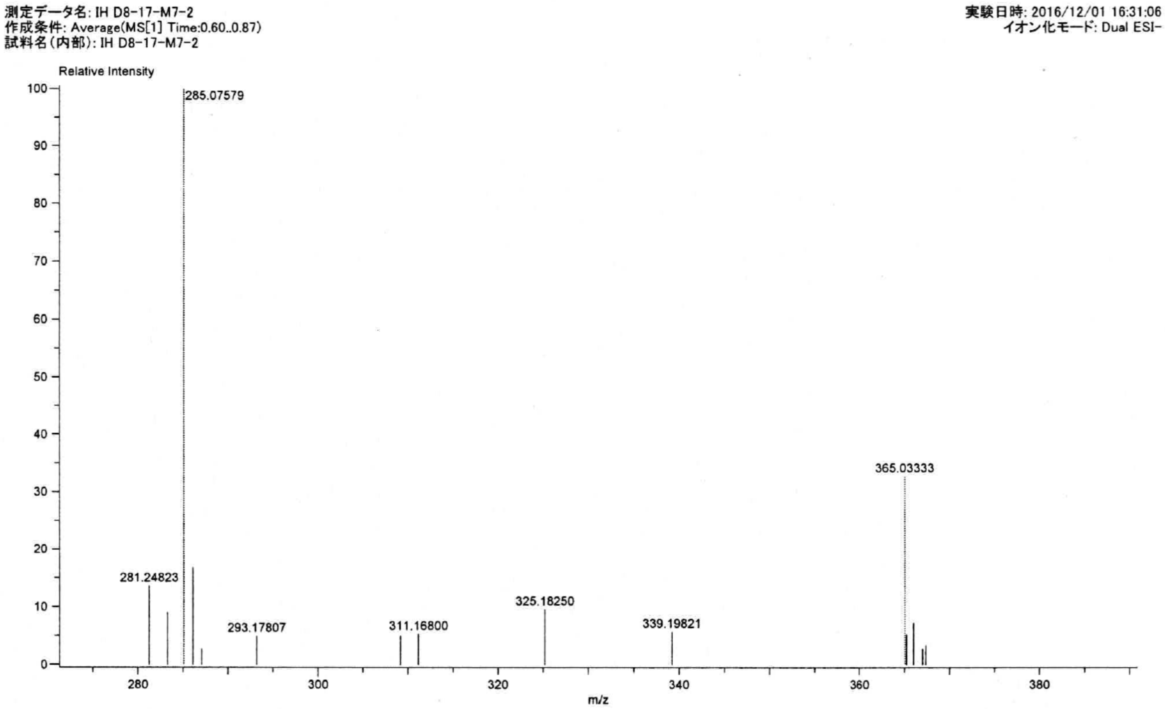

Figure 16. HRESIMS of compound **7**
